# Immunogenicity of a vaccinia virus-based severe acute respiratory syndrome coronavirus 2 vaccine candidate

**DOI:** 10.3389/fimmu.2022.911164

**Published:** 2022-07-22

**Authors:** Shan Mei, Zhangling Fan, Xiaoman Liu, Fei Zhao, Yu Huang, Liang Wei, Yamei Hu, Yu Xie, Liming Wang, Bin Ai, Chen Liang, Fengwen Xu, Fei Guo

**Affiliations:** ^1^ NHC Key Laboratory of Systems Biology of Pathogens, Institute of Pathogen Biology, and Center for AIDS Research, Chinese Academy of Medical Sciences and Peking Union Medical College, Beijing, China; ^2^ Department of Medical Oncology, Beijing Hospital, Beijing, China; ^3^ Lady Davis Institute, Jewish General Hospital, McGill University, Montreal, QC, Canada

**Keywords:** SARS-CoV-2, COVID-19, vaccine, vaccinia virus, spike protein

## Abstract

Severe acute respiratory syndrome coronavirus 2 (SARS-CoV-2) vaccines provide essential tools for the control of the COVID-19 pandemic. A number of technologies have been employed to develop SARS-CoV-2 vaccines, including the inactivated SARS-CoV-2 particles, mRNA to express viral spike protein, recombinant spike proteins, and viral vectors. Here, we report the use of the vaccinia virus Tiantan strain as a vector to express the SARS-CoV-2 spike protein. When it was used to inoculate mice, robust SARS-CoV-2 spike protein-specific antibody response and T-cell response were detected. Sera from the vaccinated mice showed strong neutralizing activity against the ancestral Wuhan SARS-CoV-2, the variants of concern (VOCs) B.1.351, B.1.617.2, and the emerging B.1.1.529 (omicron). This finding supports the possibility of developing a new type of SARS-CoV-2 vaccine using the vaccinia virus vector.

## Introduction

Severe acute respiratory syndrome coronavirus 2 (SARS-CoV-2) caused more than 257 million infections and 5 million deaths worldwide until November 23, 2021 (1). Vaccines have been the most effective tools so far to manage and control the COVID-19 pandemic. A number of technologies have been used to develop SARS-CoV-2 vaccines. These include inactivated SARS-CoV-2 vaccines, live attenuated vaccines, mRNA or DNA vaccines expressing SARS-CoV-2 spike protein or the receptor-binding domain (RBD), subunit vaccines (recombinant RBD protein or virus-like particles), and viral vector-based vaccines (2–12). The viral vector vaccines express SARS-CoV-2 antigens and are based on adenovirus, vesicular stomatitis virus (VSV), yellow fever virus 17D, Newcastle disease virus, parainfluenza virus 5, rabies virus, influenza A virus, and vaccinia virus (VACV) (13–25).

At present, the widely used COVID-19 vaccines include mRNA vaccines (mRNA-1273 and BNT162b2), inactivated virus vaccines (CoronaVac, BBIBP-CorV, and COVAXIN), and adenoviral vector vaccines expressing SARS-CoV-2 spike protein (ChAdOx1-S (Vaxzevria), ChAdOx1-S (COVISHIELD), and Ad26.COV2-S) (6–8, 13–15, 26). These vaccines have already played important roles in preventing SARS-CoV-2 infection, decreasing the severity and the fatality of COVID-19. Despite this, challenges still exist in the development and implementation of COVID-19 vaccines. One challenge is the constant emergence of SARS-CoV-2 variants of concern (VOCs). VOCs alpha (B.1.1.7), beta (B.1.351), gamma (P.1), delta (B.1.617.2), and the emerging omicron (B.1.1.529) (27) often exhibit higher transmissibility and greater pathogenicity and are less responsive to the current COVID-19 vaccines, which were designed on the basis of the ancestral SARS-CoV-2 strain. Preclinical studies also showed less protection by these vaccines in the upper respiratory tract than in the lower respiratory tract (12, 15, 22). It is pivotal to develop COVID-19 vaccines that are able to protect against different VOCs and elicit persistent protective immunity. The combination of different vaccines, the inclusion of multiple SARS-CoV-2 antigens, and the exploration of new vaccine technologies are expected to provide solutions to these challenges.

VACV has been widely used as a vaccine vector and in gene therapy (28–32). The VACV Tiantan strain was used as a smallpox vaccine in China and played a crucial role in the eradication of the local smallpox epidemic (33). Because of its safety profile in vaccination, the VACV Tiantan strain has been used as a vaccine vector for various infectious diseases, and its oncolytic property prompts its application in cancer therapy (28, 34–41). The VACV Tiantan strain has been tested in the development of vaccines for influenza virus, Ebola virus, SARS-CoV, hepatitis B virus (HBV), hepatitis C virus (HCV), and HIV-1 (35–41).

In the present study, we aimed to engineer a VACV Tiantan strain expressing SARS-CoV-2 spike protein and evaluate its effectiveness as a new SARS-CoV-2 vaccine candidate through stimulating both humoral and cellular immune responses. The mice immunized with VACV Tiantan vector-based vaccine produced high levels of SARS-CoV-2 spike protein-specific antibodies, neutralizing antibodies (NAbs), and T-cell responses, which supports the potential use of this VACV recombinant as a SARS-CoV-2 vaccine.

## Results

### Generation of vaccinia virus recombinants expressing SARS-CoV-2 spike or receptor-binding domain

**Figure 1:**
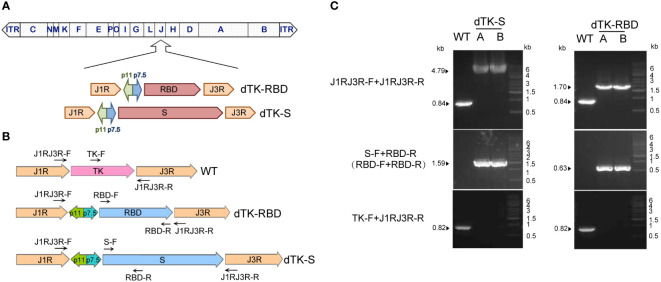
Construction of VACV recombinants expressing SARS-CoV-2 spike protein or RBD. **(A)** Schematic representation of VACV dTK-S and dTK-RBD. The SARS-CoV-2 S (with last 19aa deleted, dTK-S) and RBD sequences (319aa–529aa, dTK-RBD) were inserted into thymidine kinase (TK) locus of VACV Tiantan strain under the control of the early/late promoter p7.5 by homologous recombination. **(B)** Schematic representation of primers used for plaque purification and PCR verification. **(C)** PCR analysis of recombinant viruses confirmed the substitution of spike or RBD for the TK region. VACV, vaccinia virus; SARS-CoV-2, severe acute respiratory syndrome coronavirus 2; RBD, receptor-binding domain; dTK-S, vaccinia virus-expressing spike protein; dTK-RBD, vaccinia virus-expressing receptor-binding domain; TK, thymidine kinase.

To generate and test the VACV Tiantan strain-based COVID-19 vaccine candidate, we performed homologous recombination to insert SARS-CoV-2 spike protein (with the last 19 amino acids deleted) or the RBD sequence into the thymidine kinase (TK) locus in the VACV Tiantan strain under the control of the early/late promoter p7.5 (**Figure 1A**). Recombinant viruses were selected by PCR using primers targeting spike protein and RBD (**Figure 1B**) and further purified by five rounds of plaque selection. DNA was extracted from the purified recombinant viruses, amplified by PCR, and verified by sequencing. Primers recognizing spike protein and RBD sequences or sequences surrounding the TK region were designed for PCR (**Figure 1B**). PCR analysis and further DNA sequencing confirmed the insertion of SARS-CoV-2 spike protein or RBD sequence in the genome of the recombinant viruses (**Figure 1C**).

### Expression of receptor-binding domain and spike protein in cells infected with vaccinia virus recombinants

**Figure 2:**
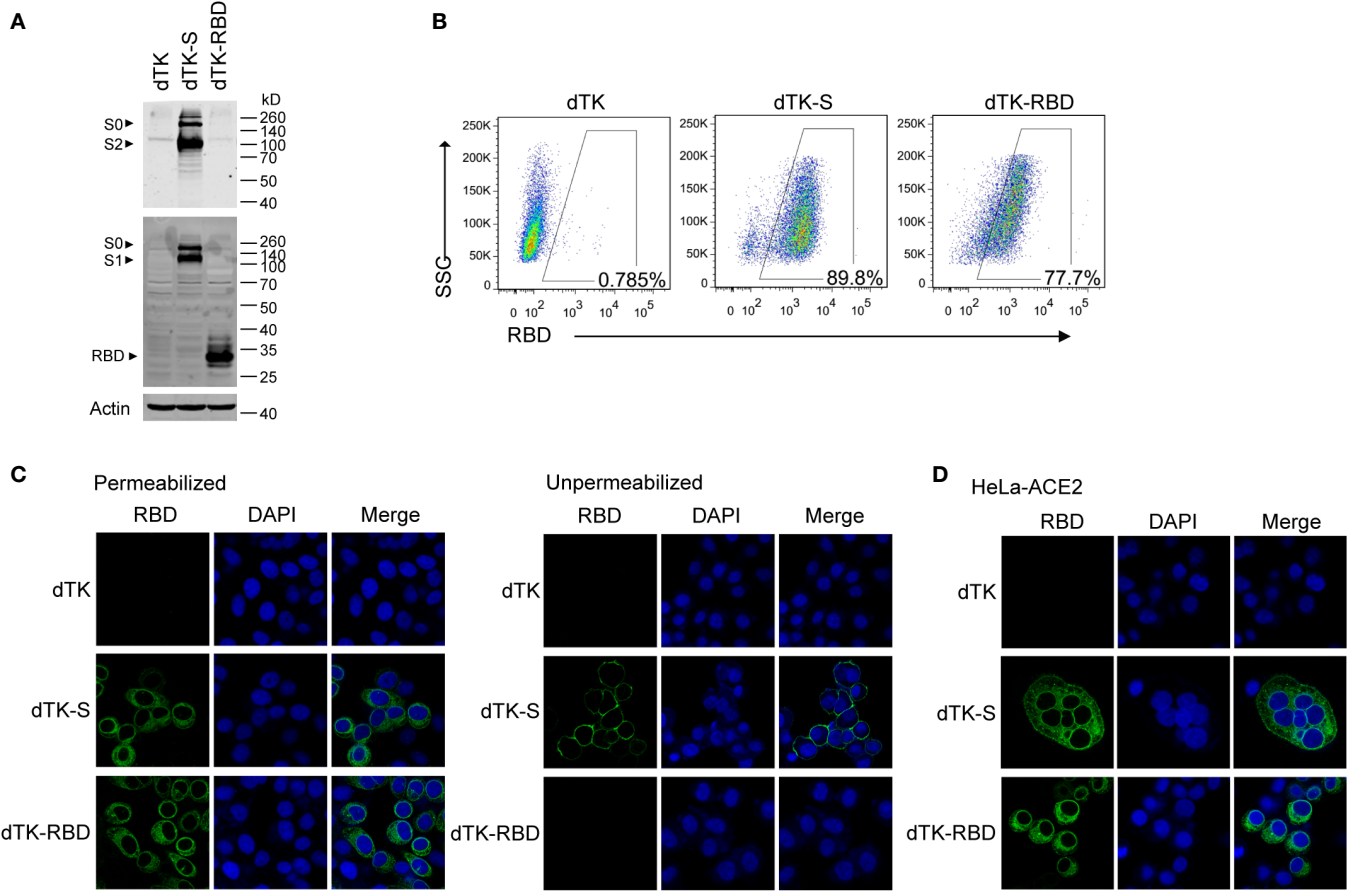
Expression of RBD and spike protein in cells infected by the recombinant VACV. **(A)** Vero cells were infected with parent VACV dTK, dTK-S, or dTK-RBD (MOI 0.01) for 24h. Cell lysates were analyzed by Western blotting using S2 or RBD-specific antibodies. **(B)** Vero cells were infected with VACV dTK, dTK-S, or dTK-RBD (MOI 0.05) for 24h. Cells were permeabilized and stained with anti-RBD antibody, followed by Alexa Fluor 488-conjugated donkey anti-rabbit antibody incubation. Percentages of FITC-positive cells were shown. **(C)** Immunofluorescence staining of HeLa cells infected with VACV dTK, dTK-S, or dTK-RBD (MOI 0.05) for 24h. Cells were fixed with 4% paraformaldehyde, permeabilized with 0.2% Triton X-100, or stained directly with anti-RBD antibody. Nuclei were stained by DAPI. **(D)** Immunofluorescence staining of syncytia formed by HeLa cells expressing human ACE2 (HeLa-ACE2). HeLa-ACE2 cells were infected with VACV dTK, dTK-S, or dTK-RBD (MOI 0.05) for 24 h, fixed, permeabilized, and stained with anti-RBD antibody. Nuclei were stained by DAPI. RBD, receptor-binding domain; VACV, vaccinia virus; dTK-S, vaccinia virus-expressing spike protein; dTK-RBD, vaccinia virus-expressing receptor-binding domain; MOI, multiplicity of infection; FITC, fluorescein isothiocyanate.

Vero cells were infected with VACV-expressing spike protein (dTK-S) or VACV-expressing RBD (dTK-RBD) for 24 h. The expression of spike protein and RBD was shown by Western blotting (**Figure 2A**). The full-length S (~190 kDa) and the cleaved S1 (~100 kDa) and S2 (~90 kDa) were observed in the Western blotting, confirming the successful processing of spike protein (**Figure 2A**). RBD or spike protein expression in infected cells was also confirmed by the results of flow cytometry with the anti-RBD antibody staining (**Figure 2B**). Results of immunofluorescence staining showed the cytoplasmic localization of RBD and spike protein in the infected cells. Cell surface expression of spike protein was detected in cells that were not permeabilized before immunostaining (**Figure 2C**). In agreement with the report that SARS-CoV-2 spike protein induces membrane fusion of ACE2-expressing cells, syncytia were observed for dTK-S infected HeLa-ACE2 cells but absent in the control dTK- or dTK-RBD-infected cells (**Figure 2D**). These data demonstrate that the engineered VACV expresses SARS-CoV-2 spike protein at the cell surface, which is fusogenic.

### Vaccinia virus dTK-S and dTK-RBD elicit high titers of spike protein-specific antibodies and T-cell responses in mice

**Figure 3:**
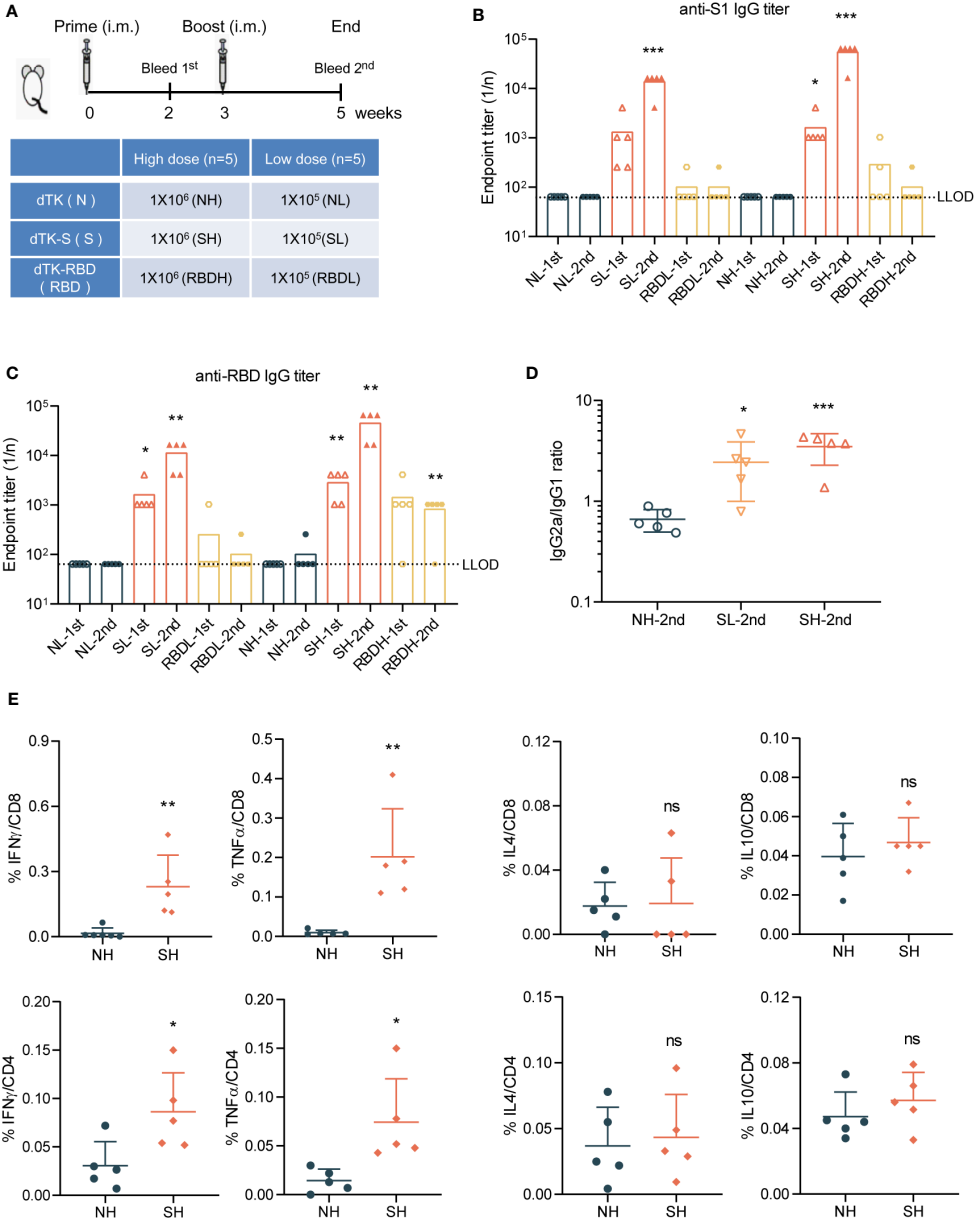
dTK-S VACV elicits effective humoral and cellular immunity in mice. **(A)** Immunization scheme. BALB/c mice aged 6–8 week were intramuscularly immunized with 1 × 10^5^ or 1 × 10^6^ PFU of control dTK, dTK-S, or dTK-RBD at week 0 and week 3. Sera were collected 2 weeks after each vaccination for further quantification of antibody responses. **(B–D)** Humoral responses in sera of immunized mice were evaluated. S1-binding antibody **(B)** and RBD-binding antibody **(C)** were analyzed by ELISA. The IgG2a/IgG1 ratio of S1-binding antibody in dTK-S immunized mice was detected by ELISA using HRP-conjugated isotype-specific antibodies **(D)**. **(E)** T-cell response induced by dTK-S. CD8 and CD4 T cells expressing IFNγ, TNFα, IL-4, and IL-10 in the spleen of dTK- or dTK-S-immunized mice (high dose) were evaluated by intracellular cytokine staining, after stimulation with S peptides. Dotted lines indicate the lower limit of detection (LLOD). Animals with a response at or below the LLOD were put on LLOD. Statistical differences as determined by Student’s *t*-tests are indicated by asterisks; *p < 0.05, **p < 0.01; ***p < 0.001. 1st, sera collected 2 weeks after prime; 2nd, sera collected 2 weeks after boost; L, low dose (10^5^ PFU); H, high dose (10^6^ PFU); N, dTK immunization; S, dTK-S immunization; RBD, dTK-RBD immunization. dTK-S, vaccinia virus-expressing spike protein; VACV, vaccinia virus; PFU, plaque-forming unit; RBD, receptor-binding domain; HRP, horseradish peroxidase.

To evaluate the antigenicity of VACV recombinants, BALB/c mice were immunized with VACV control dTK, vaccine candidates dTK-S, or dTK-RBD at doses of 10^5^ plaque-forming unit (PFU) (low dose) or 10^6^ PFU (high dose) by intramuscular route at week 0 and week 3 (**Figure 3A**). Spike protein- or RBD-binding antibodies in the sera at week 2 and week 5 were measured by ELISA (**Figures 3B, C**). The levels of spike protein/RBD-specific antibodies were higher in dTK-S-vaccinated mice than those in dTK-RBD-vaccinated mice. The high dose of 10^6^ PFU VACV immunization produced higher levels of antibodies. The dTK-RBD vaccination group exhibited no S1-binding antibody and moderate RBD-binding antibody. Levels of spike protein antibodies increased by one log 2 weeks after boosting in the dTK-S-vaccinated group. Isotype analysis of the S1-binding antibody indicated a dominant T helper 1 (Th1) cell polarization of the immune response in the dTK-S vaccination group (**Figure 3D**). To assess SARS-CoV-2 specific cellular immunity, splenocytes from dTK or dTK-S (high dose) vaccination groups were obtained and stimulated with S peptides. S-specific CD8 and CD4 T cells producing IFNγ, TNFα, IL-4, and IL-10 were measured using an intracellular cytokine staining assay. Upon S-specific stimulation, splenocytes from mice vaccinated with dTK-S produced greater numbers of IFNγ+ CD8 and IFNγ+ CD4 T cells as well as TNFα+ CD8 and TNFα+ CD4 T cells than did the control dTK group and induced a Th1-biased immune response (**Figure 3E**).

### Neutralizing antibody responses in dTK-S-vaccinated mice

**Figure 4:**
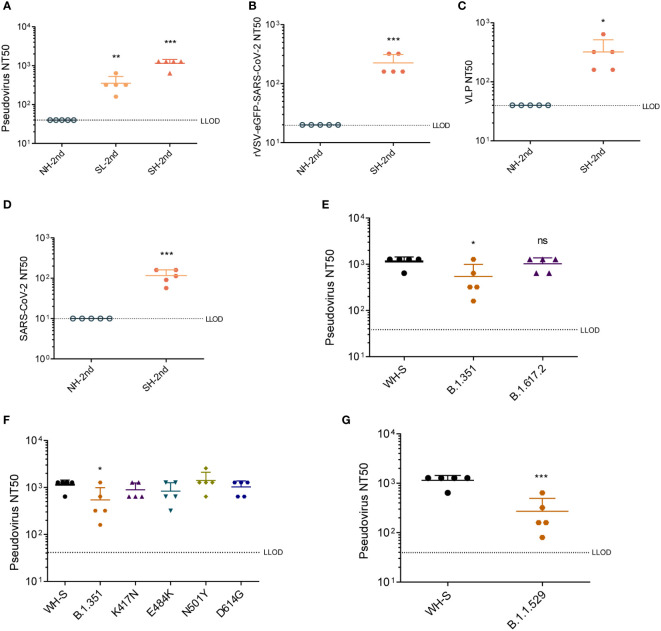
Neutralizing antibody responses in mice vaccinated by dTK-S. **(A)** Pseudovirus neutralization titers were determined for sera collected 2 weeks after dTK-S boost immunization. A 50% reduction relative to the uninfected control wells was shown as pseudovirus NT50. **(B)** Neutralization activities of the sera from the second dose of dTK-S vaccination against rVSV-eGFP-SARS-CoV-2. **(C)** VLP neutralization activities of sera from the second dose of dTK-S vaccination. **(D)** Neutralization activities of the sera from the second dose of dTK-S vaccination against SARS-CoV-2. **(E)** Neutralization activities of sera from the second dose of dTK-S vaccination for pseudoviruses carrying spike proteins of the SARS-CoV-2 Wuhan strain (WH-S) B.1.351 or B.1.617.2. **(F)** Neutralization activities of sera from the immunized mice for pseudoviruses carrying spike protein of the SARS-CoV-2 Wuhan strain (WH-S), B.1.351, or carrying single mutations K417N, E484K, N501Y, or D614G. **(G)** Neutralization activities of sera from the second dose of dTK-S vaccination for pseudoviruses carrying spike proteins of the SARS-CoV-2 Wuhan strain (WH-S) or B.1.1.529. Dotted lines indicate the lower limit of detection (LLOD). Animals with a response at or below the LLOD were put on LLOD. Statistical differences as determined by Student’s *t*-tests are indicated by asterisks; *p < 0.05, **p < 0.01; ***p < 0.001. dTK-S, vaccinia virus-expressing spike protein; VLP, virus-like particle.

Levels of NAbs were measured using the SARS-CoV-2 S-pseudovirus neutralization assay. Sera from dTK-S-vaccinated mice showed strong neutralizing activity (**Figure 4A**). We further performed a replication-competent rVSV-eGFP-SARS-CoV-2 neutralization assay (42), SARS-CoV-2 virus-like particle (VLP) neutralization assay (43), and SARS-CoV-2 neutralization assay to evaluate the NAb responses. Sera from dTK-S-vaccinated mice again showed high neutralizing activity against the rVSV-eGFP-SARS-CoV-2 (**Figure 4B**), SARS-CoV-2 VLP (**Figure 4C**), and SARS-CoV-2 live virus (**Figure 4D**). We also measured the neutralizing activity of sera from the dTK-S-vaccinated mice against VOCs B.1.351 (beta) or B.1.617.2 (delta) using pseudoviruses carrying the spike protein (417N/484K/501Y/614G) or spike protein (452R/478K/614G/681R). Sera from the dTK-S-vaccinated mice showed strong neutralizing activity against spike proteins from the ancestral Wuhan strain B.1.617.2 and moderate neutralizing activity against B.1.351 (**Figure 4E**). We further tested spike proteins carrying single mutations of 417N, 484K, 501Y, or 614G, and strong neutralization against these spike protein mutants was detected, indicating that these mutants together contribute to the poor neutralization of B.1.617.2 by the immunized mouse sera (**Figure 4F**). In light of the current dominant transmission of omicron, we measured the neutralizing activity of sera from the dTK-S-vaccinated mice against B.1.1.529 (omicron) using pseudovirus carrying the omicron spike protein. Although the neutralizing activity against B.1.1.529 decreased, all of the five mice elicited positive NAb response against the omicron variant (**Figure 4G**). Together, these data demonstrate that the dTK-S VACV elicits a strong NAb response to the ancestral Wuhan SARS-CoV-2 and VOCs B.1.351, B.1.617.2, and B.1.1.529.

## Discussion

VACV has been used as a vaccine vector for the management of infectious diseases and cancers. Several vaccinia vector-based SARS-CoV-2 vaccine candidates have been tested using a highly attenuated VACV strain Modified Vaccinia Ankara (MVA) and exhibited protection in immunized animals (22–25, 44–49). Recombinant MVA-expressing spike protein or RBD were shown to elicit strong NAb responses, produce S-specific CD8 T cells, and protect vaccinated animals against a lethal intranasal SARS-CoV-2 challenge. The VACV Tiantan strain was widely used as a smallpox vaccine in China and has been successfully adopted as a viral vector to develop vaccines against a variety of virus infections, such as influenza virus, Ebola virus, SARS-CoV, HBV, HCV, and HIV-1 (35–41). The VACV-based HIV-1 vaccine candidate has completed a phase IIa clinical trial (NCT01705223). Here we showed that the VACV Tiantan strain-based SARS-CoV-2 vaccine candidate (dTK-S) induced high levels of spike protein-specific antibodies in mice. Sera from the immunized mice exhibited neutralization activity against the ancestral Wuhan stain and VOCs including beta (B.1.351), delta (B.1.617.2), and omicron (B.1.1.529). The dTK-S virus also induced a predominantly Th1-type response to spike protein. These data support the potential of further developing dTK-S into a new SARS-CoV-2 vaccine.

Intranasal administration of vaccines based on the VACV was reported to stimulate both efficacious mucosal and systemic immune responses. For example, Xie et al. generated a recombinant VACV harboring Zaire Ebola virus glycoprotein (GP) that, when administered in mice *via* the intranasal route, induced GP-specific IgA and IgG in serum, nasal wash, and vaginal wash, as well as specific cellular immunity (37). Similarly, intranasal delivery of VACV vaccine candidates targeting the SARS-CoV-2 induced spike protein-specific IgG and IgA in serum (47, 50). The increased mucosal immune response supports the high potential of recombinant VACV to prevent mucosal infection of SARS-CoV-2. It is difficult to compare the effectiveness of vaccine candidates based on the VACV Tiantan strain and those derived from other VACV strains because these VACV vaccine candidates have been evaluated with different immunization routes including intravenous injection, intramuscular injection, intranasal injection, intraperitoneal injection, and different vaccination strategies such as prime only or prime-boost, and low or high injection doses (22, 24, 47, 51). Further research is warranted to characterize in detail the impact of different immunization routes and vaccination strategies on the effectiveness of VACV Tiantan candidates.

It was reported that VACV-induced antibodies remained stable for 1 to 75 years after vaccination, and VACV-specific cellular responses declined with a half-life of 8 to 15 years (52). The level of RBD-specific IgG in RBD-expressing VACV immunized mice sustained for 195 days post-immunization (53), further supporting the long-lasting immune response elicited by VACV-based vaccines.

We found that the dTK-RBD did not induce as strong humoral immunity as the dTK-S despite the high expression of RBD in the infected cells. dTK-S may present more epitopes for antibody production, or membrane-associated spike protein exhibits stronger immunogenicity than soluble RBD. Membrane-anchored immunogens have been shown to elicit greater immune responses than cytosolic expressions in an MVA vector (54, 55), and recombinant MVA RBD encoding a membrane-bound RBD showed higher immunogenicity than soluble RBD (25). Recombinant VACV Tiantan expressing the membrane-bound RBD may increase its immunogenicity, but further studies are needed to test this hypothesis.

In conclusion, we have generated a SARS-CoV-2 vaccine candidate using the VACV Tiantan strain. Mice immunized with dTK-S mount robust humoral immune responses to spike protein of both the ancestral SARS-CoV-2 and the circulating VOCs. Meanwhile, this vaccine candidate also induced S-specific T-cell responses, thus holding promise as another tool to be added to the COVID-19 vaccine arsenal.

## Materials and methods

### Construction and purification of vaccinia virus recombinants

Codon-optimized cDNA clones encoding SARS-CoV-2 spike (with C-terminal 19 amino acids deletion) or the RBD were synthesized by Tsingke Biotechnology Co., Ltd. (Beijing, China). Spike protein and RBD sequences were inserted into the thymidine kinase (TK) locus of the VACV Tiantan strain under the transcriptional control of the early/late promoter p7.5 by homologous recombination. After five rounds of plaque purification, insertion of spike or RBD into the viral genome was confirmed by PCR and DNA sequencing. Purified recombinant viruses were amplified by infecting Vero cells and titrated by plaque assay. Primers used were as follows: J1RJ3R-F, CAGATTTCTCCGTGATAGGT; J1RJ3R-R, CTTAGTAAATCCGCCGTACT; S-F, GGATCCATGTTCGTTTTCCTTG; RBD-F, CGCGTCCAGCCAACC; RBD-R, CTTCTTGGGACCGCATAC; TK-F, GAAGGACAGTTCTTTCCAGACATTGTTG.

### Western blotting

Vero cells were infected with VACV recombinants expressing SARS-CoV-2 spike protein or RBD. Cell lysates were harvested 24 h post-infection; samples were boiled for 10 min, separated in a 10% sodium dodecyl sulfate–polyacrylamide gel electrophoresis (SDS-PAGE) gel, and transferred onto nitrocellulose filter membranes. After being blocked with 5% milk, membranes were probed with the antibodies anti-SARS-CoV-2 S2 (GTX632604, GeneTex, Irvine, CA, USA), anti-SARS-CoV-2 RBD (Sino Biological Inc., Beijing, China; 40592-T62), and anti-actin (Sigma-Aldrich, St. Louis, MO, USA; A1978) followed by incubation with IRDye 800-labeled IgG and IRDye 680-labeled IgG secondary antibodies (Li-Cor Biosciences, Lincoln, NE, USA). Protein signals were scanned with Odyssey (Li-Cor Biosciences, Lincoln, NE, USA).

### Flow cytometry

dTK-S- or dTK-RBD-infected Vero cells were permeabilized with 0.1% Triton X-100 and incubated with the anti-RBD antibody for 1 h, followed by incubation with Alexa Fluor 488-conjugated donkey anti-rabbit antibody (Thermo Fisher Scientific, Waltham, MA, USA; A11034). Samples were analyzed with FACS Canto II (BD Biosciences, San Jose, CA, USA).

### Immunofluorescence assay

HeLa cells were infected with VACV recombinants expressing SARS-CoV-2 spike protein or RBD for 24 h and fixed with 4% paraformaldehyde for 10 min. Cells were permeabilized with 0.2% Triton X-100 for 10 min. Permeabilized and un-permeabilized cells were blocked with 5% bovine serum albumin (BSA) and then incubated with the anti-RBD antibody for 1 h, followed by incubation with Alexa Fluor 488-conjugated donkey anti-rabbit antibody (Thermo Fisher Scientific, A11034) for 1 h. Nuclei were stained by DAPI. Images were captured with an inverted confocal microscope Leica TCS SP5 (Leica Microsystems, Wetzlar, Germany).

### Immunization of animals

All animal experiments were performed in accordance with guidelines and regulations and approved by the Institutional Animal Care and Use Committee of Beijing Protein Innovation Co., Ltd. Female BALB/c mice aged 6 to 8 weeks were intramuscularly immunized with 1 × 10^5^ and 1 × 10^6^ PFU of dTK, dTK-S, or dTK-RBD at week 0 and week 3, respectively. All animals were euthanized following the animal use protocols 2 weeks after the last immunization. Sera were collected 2 weeks after each vaccination for further quantification of antibody responses.

### Detection of S1/RBD-binding antibodies by ELISA

S1-binding antibodies were detected with SARS-CoV-2 Spike S1 Antibody Titer Assay Kit (Mouse) (Sino Biological Inc., KIT007). RBD-binding antibodies were detected with Spike RBD Antibody Titer Assay Kit (Mouse) (Sino Biological Inc., KIT006) according to the instructions of the manufacturers. Briefly, sera were serially 4-fold diluted in dilution buffer, and 100 μl of the diluted sera was added to spike S1/RBD precoated microplates and incubated for 2 h at room temperature. After the microplates were washed, diluted horseradish peroxidase (HRP)-Rabbit anti-Mouse IgG was added to the plates and incubated for 1 h at room temperature. The plates were then incubated with substrate solution for 20 min. Levels of antibodies were determined by measuring the optical density (OD) at 450 nm with a microplate reader after termination with stop solution. Final end point titers (1/n) were defined as the highest dilution that yielded an absorbance >0.1. For detection of antibody isotypes, HRP-conjugated Goat anti-Mouse IgG1 (Abcam, Cambridge, UK; ab97240) and HRP-conjugated Goat anti-Mouse IgG2a (Abcam, ab97245) were used.

### Intracellular cytokine staining

For intracellular cytokine staining, splenocytes were collected and stimulated with S_268-276_ (GYLQPRTFL), S_233-247_ (INITRFQTLLALHRS), S_421-437_ (YNYKLPDDFTGCVIAWN), and S_442-458_ (DSKVGGNYNYLYRLFRK) peptides for 1 h. Brefeldin A was added, and cells were incubated for an additional 5 h. After stimulation, surface staining was performed using anti-CD3-FITC (BioLegend, San Diego, CA, USA; 100204), anti-CD4-PerCP/Cy5.5 (BioLegend, 100434), and anti-CD8-PE/Cy7 (BioLegend, 100722). Then cells were fixed, permeabilized, and stained with anti-IFN-γ-PE (BioLegend, 163504) and anti-IL-4-APC (BioLegend, 504106). Data were acquired by FACS Canto II (BD).

### Pseudovirus neutralization assay

Pseudoviruses were produced by co-transfection 293T cells with psPAX2 encoding the structural polyproteins Gag-Pol, pLenti-GFP containing the GFP and luciferase reporters, and cDNAs encoding SARS-CoV-2 spike protein (last 19aa deleted) or the indicated mutants. Supernatants were harvested at 48 h post-transfection and passed through 0.45-μm filters. Twofold serial dilutions of the heat-inactivated sera were mixed with the above pseudoviruses for 1 h at 37°C and then added to HeLa-ACE2 cells in a 96-well plate. The luciferase activities were determined using the Steady-Glo Luciferase Assay System (Promega, Madison, WI, USA; E2520) 48 h post-infection. The neutralization titers were defined as the reciprocal of the highest dilution at which a 50% reduction was observed relative to the uninfected control wells.

### rVSV-eGFP-SARS-CoV-2 neutralization assay

Replication-competent recombinant VSV-SARS-CoV-2 viruses (rVSV-eGFP-SARS-CoV-2) in which VSV-G was substituted with the SARS-CoV-2 S protein were gifts from Dr. Aihua Zheng (42). Twofold serial dilutions of the heat-inactivated sera were mixed with rVSV-eGFP-SARS-CoV-2 at 37°C and then added to 293T-ACE2 cells in a 96-well plate. The GFP-positive cells were calculated by flow cytometry 24 h post-infection. The neutralization titers were determined as the highest dilution at which a 50% reduction was observed.

### VLP neutralization assay

SARS-CoV-2 VLPs were produced by co-transfecting 293T cells with CoV2-N, CoV2-M-IRES-E, CoV-2-Spike, and Luc-PS9, which were gifts from Jennifer Doudna (Addgene, Cambridge, MA, USA; plasmid # 177950, 177938, 177939, and 177942) (43). Supernatants were harvested at 48 h post-transfection and passed through 0.45-μm filters. Twofold serial dilutions of the heat-inactivated sera were mixed with the above VLPs for 1 h at 37°C and then added to 293T-ACE2 cells in a 96-well plate. The luciferase activities were determined using the Steady-Glo Luciferase Assay System (Promega, E2520) 24 h post-infection. The neutralization titers were determined as the highest dilution at which a 50% reduction was observed relative to the uninfected control wells.

### SARS-CoV-2 neutralization assay

Twofold serial dilution of the heat-inactivated sera was mixed with 100 TCID_50_ SARS-CoV-2 (WH-09, GenBank: MT093631) for 1 h at 37°C in a 5% CO_2_ incubator and then added to Vero cells in 96-well plates. The plates were incubated for 3–5 days to observe cytopathic effect (CPE). The serum dilution at which 50% of the cells were protected against infection was calculated.

### Statistical analyses

Each measurement represents a single animal. Statistical differences are determined by two-tailed Student’s *t*-tests; *p < 0.05, ** *p* < 0.01; ***p < 0.001.

## Data availability statement

The original contributions presented in the study are included in the article/supplementary material. Further inquiries can be directed to the corresponding authors.

## Ethics statement

The animal study was reviewed and approved by the Institutional Animal Care and Use Committee of Beijing Protein Innovation Co., Ltd.

## Author contributions

FG, FX, FZ, and BA conceived the project. FX, SM, FZ, XL, ZF, YH, LWe, YMH, YX, and LWa performed the experiments. CL, FX, and FG wrote the manuscript. All authors reviewed the manuscript and discussed the work. All authors contributed to the article and approved the submitted version.

## Funding

This study was supported by funds from CAMS Innovation Fund for Medical Sciences (CIFMS 2021-1-I2M-038), the National Key Plan for Scientific Research and Development of China (2018YFE0107600 and 2022YFE020378), the Ministry of Science and Technology of China (2018ZX10301408-003), the National Natural Science Foundation of China (82072288), and the Canadian Institutes of Health Research (CCI-132561).

## Conflict of interest

FG, FX, SM, and ZF are inventors on pending and issued patents on SARS-CoV-2 vaccines.

The remaining authors declare that the research was conducted in the absence of any commercial or financial relationships that could be construed as a potential conflict of interest.

## Publisher’s note

All claims expressed in this article are solely those of the authors and do not necessarily represent those of their affiliated organizations, or those of the publisher, the editors and the reviewers. Any product that may be evaluated in this article, or claim that may be made by its manufacturer, is not guaranteed or endorsed by the publisher.
